# *Polycladia myrica*-based delivery of selenium nanoparticles in combination with radiotherapy induces potent *in vitro* antiviral and *in vivo* anticancer activities against Ehrlich ascites tumor

**DOI:** 10.3389/fmolb.2023.1120422

**Published:** 2023-04-12

**Authors:** Sahar E. Abo-Neima, Abdelhamid A. Ahmed, Mostafa El-Sheekh, Mofida E. M. Makhlof

**Affiliations:** ^1^ Physics Department, Faculty of Science, Damanhour University, Damanhour, Egypt; ^2^ Plastic Surgery Department, Faculty of Medicine, Tanta University, Tanta, Egypt; ^3^ Botany Department, Faculty of Science, Tanta University, Tanta, Egypt; ^4^ Botany and Microbiology Department, Faculty of Science, Damanhour University, Damanhour, Egypt

**Keywords:** *Polycladia myrica*, selenium nanoparticles, antiviral, HCT-116, EAC, *in vivo*, *in vitro*, laser therapy

## Abstract

**Background:** Over the last few decades, nanotechnology has entered daily life through various applications, therefore, there has been a trend toward developing new approaches to green-mediated nanotechnology that encourage nanomaterial formation through biological methods such as plants or microorganisms. Algae have gained increasing attention from nanotechnology scientists and have paved the way for the emergence of “algae nanotechnology” as a promising field.

**Methods:** Via using the aqueous extract of the brown alga *Polycladia myrica*, selenium nanoparticles were synthesized and characterized by using seven instruments: SEM, TEM, UV spectra, Zeta potential, EDX, X-ray diffraction, and FTIR. *P. myrica* selenium nanoparticles (PoSeNPs) were then examined for their antiviral activity against HSV-1 (Herpes simplex I) and anticancer against human colon cancer cell line (HCT-116) *in vitro* and *in vivo* alone and in combination with laser therapy of power 2 mW against Ehrlich carcinoma (EAC).

**Results:** PoSeNPs ranging between 17.48 nm and 23.01 nm in size, and EDX revealed the selenium mass and its atoms as 0.46% ± 0.07% and 0.08% ± 0.01% respectively. Their anticancer potentiality *in vitro* was with maximum inhibitions of 80.57% and 73% and IC_50_ = 14.86 μg/mL and 50 mg/mL against HCT-116 and EAC cell lines respectively, while their *in vivo* alone and in combination with laser therapy of power 2 mW showed a potent therapy effect against Ehrlich ascites carcinoma (EAC).

**Conclusion:** This study concluded that PoSeNPs do not have a toxic effect; they exhibit high effectiveness as a photothermal agent for cancer therapy, with promising applications in future biomedical fields. The combined therapy showed a significant decrease in tumor volume, massive tumor cell necrosis, shrinking, and disappearance. It also showed improvement in liver TEM, histology, kidney function: urea and creatinine, and liver enzymes: ALT, and AST.

## Introduction

Nanoscience has witnessed rapid advancements in various fields and has been applied to numerous critical medical and industrial applications (El-Sheekh, et al., 2022). Over the last few decades, nanotechnology has entered daily life through various applications (Panda et al., 2020). This has helped to open possibilities to create new tools that can boost the effectiveness, toxicity, environment-friendliness, and biohazardousness of synthesized nanoparticles, given that traditional industrial methods require complicated mechanisms by using high temperatures and pressures that cause environmental hazards (Elshahawy, et al., 2018). Therefore, there has been a trend toward developing new approaches to green-mediated nanotechnology that encourage nanomaterial formation through biological methods such as plants or microorganisms (El-Sheekh et al., 2021a). Algae gained increasing attention from nanotechnology scientists, and paved the way for the emergence of “algae nanotechnology” as a promising field. It is referred to as phyco-nanotechnology since algae are widespread and photoautotrophs (Khalid, 2019; Bhuyar et al., 2020; Rahman et al., 2020; Ingle et al., 2021; El-Sheekh et al., 2022). Algae can biosynthesis nanoparticles, either intracellularly or extracellularly, depending on their cellular reactions, secondary metabolites, or extraction of algal contents. This can be achieved through their capability to reduce hyperaccumulating metal ions and convert them to stable forms, which accordingly helps regulate the synthesis of environmentally safe and effective nanoparticles with various characteristics (El-Sheekh et al., 2022; Hanna et al., 2022). They help in reducing and capping the produced nanoparticles at normal room temperature and pressure. Moreover, they are not hazardous to use in a variety of applications that achieve a greener lifestyle with green nanoscience (El-Sheekh et al., 2021b; Cai et al., 2021; Yosri et al., 2021; El-Sheekh, et al., 2022).

Macroalgae are large eukaryotic multicellular organisms that can be classified into three main groups: green Chlorophyta, red Rhodophyta, and brown Phaeophyta (Amlund et al., 2019). *Ulva Lactuca* and *Hypnea musciformis* aqueous extracts were utilized as reductant sources for Ag-NPs with a size of 40–65 nm and a spherical shape exhibiting promising antifungal action against numerous pathogenic fungi (Chugh et al., 2021). Algal-mediated nanoparticles are growing increasingly popular in biotechnological and biomedical fields such as tumor treatment, drug delivery enhancement, coagulant agents, and treatment of microorganisms (Khan et al., 2019; Dalal and Biswas, 2020; Panda et al., 2020). Fouda et al. (2022) synthesized spherical MgO-NPs with a size of 3–18 nm by using *Cystoseira crinite* extract. Gu et al. (2018), meanwhile, synthesized spherical CuO-NPs with a size of 6–7.8 nm by using *Cystoseira trinodis* extract. According to Algotiml et al. (2022), the extracts of three macroalgae, namely *Ulva rigida, Cystoseira myrica,* and *Gracilaria foliifera*, were used to biosynthesize Ag-NPs with promising anticancer activity against breast cancer cell lines. However, there is more concentrated interest focused on selenium nanoparticles (SeNPs) because of their promising applications and distinctive properties (Cui, et al., 2016; Hussein, et al., 2019b).

Selenium (Se)—one of the essential minor elements abundant in both the earth’s crust and the environment in organic and inorganic redox states—has many beneficial effects on human health, including acting as an antioxidant and preventing cancer initiation, growth, and metastasis without causing toxic side effects (Hariharan and Dharmaraj, 2020). On the other hand, Se and its states have a wide variety of biological availability and activities. Because of their high bioavailability and diverse biological activity, Se nanoparticles (SeNPs) are widely used in biomedicine. They have several biological activities such as anticancer and antimicrobial activities. For example, they hold promising antibacterial activity against *Staphylococcus aureus* when compared with Ampicillin, which is a commercial drug (Huang, et al., 2003; Srivastava and Mukhopadhyay, 2015; Baozhen et al., 2017; Hussein, et al., 2019a; Darwesh, et al., 2019). Cancer is a fatal disease that occurs when cells proliferate abnormally in any body organ. Aside from the high cost of anticancer treatments and their damaging side effects on the whole body, there is difficulty in finding effective drugs that treat various types of cancer. All of these issues contribute to the need to develop a new and effective therapy (Patel et al., 2020). Chemotherapy and radiotherapy are the two most effective forms of cancer treatment; however, they cause fatal side effects. Thus, there has been a search for an alternative method to decrease harmful treatments, and nanoparticles have recently been used in cancer treatment (Health and Davis., 2008). Cancer cells are highly prooxidant-susceptible, and high doses of Se cause noticeable toxic effects. However, Se-containing drugs have been mostly used against aggressive late-stage cancers (Fernandes and Gandin, 2015). Compared to other Se derivatives, the new Se species: SeNPs show lower and higher toxicity and biocompatibility, respectively, for their application as therapeutic agents, particularly in cancer treatment. Unlike large-scale nanoparticles, the severely small size helps nanoparticles penetrate the cells of mammals through phagocytosis or endocytosis. The metabolic roles of Se within cells comprise the catalysis of several antioxidant enzyme active sites, including thioredoxin reductase, glutathione reductase, and glutathione peroxidase (Flora et al., 2002; Hamouda et al., 2019; Liu et al., 2019; Cláudio et al., 2021; Hassan et al., 2021).

Viruses that are made up of genetic material, i.e., DNA or RNA, are small particles encased in a protein coat. They cause infection in a specific manner that replicates only within host cells. Nanoparticles have the ability to adhere to and penetrate the glycoprotein found on the surface of the virus, causing glycoprotein agglutination and preventing binding between the virus and the host cell. Consequently, this prevents viral entry into the host cell, or can cause viral genetic materials multiplication inhibition and RNA action blocking (Gurunathan et al., 2020; Ratan et al., 2021). As observed, only a few investigations proved the phycosynthesizing of selenium nanoparticles, despite their mentioned biological importance. Makhlof et al. (2022) synthesized SeNPs by using *Ulva lactuca* extract, giving promising results in cancer treatment. Meanwhile, Touliabah et al. (2022) synthesized SeNPs by using *Polycadia myrica* extract, showing promising antiviral and anticancer activities.

For the first time, this study aims to test the previously synthesized *Polycladia myrica* SeNPs (Touliabah, et al., 2022) *in vitro* against HepG2 carcinoma cell lines, *in vivo* against Ehrlich carcinoma alone, and with radiation laser therapy. This is to investigate the impact of its combined therapy by using Ehrlich carcinoma bearing mice as a tumor model. The study also focuses on the antiviral activity of PoSeNPs against Herpes simplex I.

## Materials and methods

### PoSeNPs synthesis and characterization

As previously mentioned in Touliabah, et al., 2022, *P. myrica* was first sampled, and then identified. After the samples were cleaned, dried, and powdered, they were stored for further use. According to Hashemi et al. (2015), the algal aqueous extract was done, and then filtrated and stored at 4°C (Thamer et al., 2018). In order to determine the active groups in *P. myrica* aqueous extract that are responsible for nanoparticles being phycosynthesized, FTIR (FT/IR-6100 type A) was used with spectra between 4,000 nm^-1^ and 400 nm^-1^. PoSeNPs were synthesized according to Touliabah, et al. (2022); Abdelhamid et al. (2022). They were detected by UV–Vis Spectrophotometer: Thermo Scientific Evolution TM 300, Thermo Fisher Scientific, Waltham, MA, United States of America, in a range of 200–800 nm. Solution centrifugation was then conducted at 4,400 g for half an hour by washing the produced nanoparticles with double distilled water and pure ethanol (Vikneshan, et al., 2020). The produced NPs were then dried at 50°C and stored in an airtight container for further utilization studies. The shape and size of nanoparticles were detected by using TEM and SEM—JEM-2100 (JEOL Ltd., Tokyo, Japan) and JSM-6490LV (JEOL Ltd., Tokyo, Japan) respectively. SeNPs EDX study was conducted by using the latter between 0 and 12 keV. XRD spectrum was used to study the crystallinity and elementary of nanoparticles at 30 kV and 10 mA with 2.2 KW Cu anode radiation by using XRD-6000 detector (Shimadzu Corp., Kyoto, Japan). The identification of the algal extract biomolecules functional groups was made through FTIR, which can make the reduction and capping for SeNPs in the range of 400–4,000 cm^−1^ by using FTIR spectroscopy. Meanwhile, the effective surface charges of PoSeNPs at various variables and its long-term stability were estimated by using Zeta Analyzer (ZetaPlus, Brookhaven Instruments, Holtsville, NY, United States).

### Cytotoxicity and antiviral activity test of PoSeNPs

In a previous study (Touliabah et al., 2022), the PoSeNPs cytotoxicity was examined by using Vero cells extracted from an African green monkey kidney (ATCC, Manassas, VA, United States) and they were provided by the American Type Culture Collection (ATCC). Dulbecco’s Modified Eagle’s Medium (DMEM) was used as a growing medium for such a cell line (Vijayan et al., 2004) through MTT assay (Mosmann, 1983; Gomha et al., 2015). In confluent Vero cells in this study, the herpes simplex type-1 virus (HSV-1) was used for this test (Randazzo et al., 2017). By using the Spearman-Karber method, a number of propagated viruses in eight wells supplied by 20 µL of inoculum in each was calculated by a dose of infectious tissue culture (TCID50) (Pinto et al., 1994).

### Cytotoxicity of PoSeNPS

#### *In vitro* anticancer activity by using the viability assay against HCT-116 and EAC cell lines

The EAC and human colon cancer cell line (HCT-116) in this study were obtained from the National Cancer Institute, Cairo, Egypt, and the American Type Culture Collection (ATCC, Rockville, MD, United States) respectively. Meanwhile, the supplied fetal bovine serum, trypan blue dye, MTT, and dimethyl sulfoxide (DMSO) were obtained from Sigma-Aldrich (St. Louis, MO, United States). Lonza Group AG (Bornem, Belgium) offered the HEPES buffer solution, RPMI-1640, L-glutamine, 0.25% trypsin-EDTA, and gentamicin. The RPMI-1640 media—with 10% inactivated fetal calf serum and 50% gentamicin—was used for the growth of used cell lines. After that, it was incubated in 37°C humid atmosphere with 5% carbon dioxide and then subcultured about 2–3 times weekly. The tumor cells were suspended in the medium at 5 × 10^4^ cells/well concentration with the use of Corning 96-well tissue culture plates for anticancer test and incubated for a whole day. The concentrations of PoSeNPs (1.56–25) were then placed onto the used plates in three duplicates with six wells of 0.5% DMSO as a control. The MTT assay determines the number of viable cells after a whole day of incubation. In each well, the RPMI 1640 medium without phenol red was replaced with 100 µL of fresh culture medium for 96-well plates, and 10 μL of the stock solution of 12 mM MTT (5 mg of MTT in 1 mL of PBS) was set to each well that included the untreated controls. The plates were then incubated for 4 hours at 37°C with 5% carbon dioxide. Fifty µl of DMSO in each well were then mixed with a pipette carefully, and the plates were incubated for 10 min at 37°C through the following formula:

$$\left[\left(OD_{t}/OD_{c}\right)\right]\times 100\%$$

The number of viable cells was calculated, where OD_t_ is the mean optical density of PoSeNPs treated wells, and OD_c_ is the mean optical density of untreated cells at 590 nm with a microplate reader (SunRise, Tecan Group Ltd., Männedorf, Switzerland). The 50% inhibitory concentration (IC_50_) was computed by using GraphPad Prism software (San Diego, CA, United States) with a dose-response curve for each amount graphic plot (Mosmann, 1983).

#### Microscopic observation of HCT-116 cell lines treated with PoSeNPs

For microscopic observation, the plates were inverted to remove the excess medium, followed by washing the wells three times with 300 µL of phosphate-buffered saline (pH 7.2). For cell fixation, 10% formalin was used for a quarter of an hour at room temperature. After that, the cell staining was done for 20 min with 100 µL crystal violet with 0.25% concentration. Deionized water was used to remove the excess stain and drying for the plates. The changes compared to control cells were observed by using an Olympus inverted microscope with a digital microscopy camera CKX41 (Olympus Corp., Tokyo, Japan) at a magnification of ×200.

### *In vivo* anticancer activity by using EAC cell line

#### Tumor induction and volume determination

The intramuscular injection method, with 1 × 10^6^ viable EAC cells, was used to obtain solid tumors with 0.2 mL in the right thigh of the lower limb of each mouse. After 10 days of injection, mice with a palpable solid tumor with a diameter of 10 mm³ were used in this study. Tumor volume was determined by using the standard solid tumor formula: V = 1/2 ∗ (D ∗ d_2_) (Goto et al., 2002)— where *V*, *D*, and *d* are the tumor volume, the tumor’s higher diameter, and the tumor’s lower diameter, respectively.

#### Laser and PoSeNPs therapy

##### Exposure facility system

A laser treatment unit (Mustang, 2000; Germany)—with low intensity, a maximum power of 2 MW, emission frequencies ranging from 10 to 3,000 Hz, and two outputs—was used to allow simultaneous connection of two laser emitters. As the mice were anesthetized, ether was used to remove the hair that covers the tumor. Securing the mice was done on a cork plate with the tumor raised, and the laser probe was tightly inserted for tumor laser irradiation for 10 min.

##### Experimental design

Fifty male Swiss albino mice—nearly 8 weeks of age with body weights ranging between 20 and 25 g—were used. They were obtained from the Egyptian Organization for Biological Products and Vaccines (VACSERA). Tumor induction was done for all used mice with a volume of 0.7–1 cm^3^. The mice were then assigned randomly into five groups—each of which had 10 mice. They are Group 1: Non-Tumor Bearing Mice (NTBM), which was used as a negative control with 0.5 mL saline/day orally; Group 2: Tumor-Bearing Mice (TBM) without any treatment served as a positive control group; Group 3: Tumor-Bearing Mice (Laser) exposed to laser radiation therapy at 2 MW, 3000Hz for 10 min for 14 consecutive days; Group 4: (PoSeNps) TBM that received 50 mg PoSeNps/kg body weight for 14 consecutive days; and Group 5: (Laser + PoSeNps) Tumor-bearing mice were injected with PoSeNps at the same dose as Group 4, and then treated tumor with radiation as Group 3. All cages were placed in an air-conditioned room at 22 ± 3°C and humidity 50% ± 10% with 12 h light/dark cycle. All mice had free access to food and water, with careful observation of their activity daily recorded 24 h from the last injection of nano plus radiation. All mice were anesthetized with ether.

#### Tissue preparation

Mice sacrifice was done after the last treatment, the liver was divided into three parts. The first one was used for histopathological investigation by using a microscope (Trinocular biological microscope 400x–600x, United States) by storing it in 10% formalin. The second one was used for homogenization by rinsing it with saline (0.9% NaCl), and then suspended in normal saline (0.5 g tissue/5 mL saline) by using a Teflon Homogenizer. After that, the homogenates were centrifuged and the supernatant was stored at −20°C for further analysis. The third one was used for TEM by storing them in glutaraldehyde (Kamal et al., 2017).

#### Biochemical examination of serum and tissue samples

Centrifugation at 3,000 r/min for 20 min was done to obtain serum samples and then the samples were stored at −20°C storing for biochemical analysis through the estimation of AST (aminotransferase), urea, creatinine, aspartate, and ALT (alanine aminotransferase) by using commercial kits from the Biodiagnostic Company for Laboratory Services according to the received instructions. EAC pellets were collected from the ascetic fluids by centrifugation from 1,000 rpm to 2000 rpm for 10 min at 4°C and livers were homogenized in a cold buffer. The levels of glutathione (GSH, non-enzymatic antioxidant), Thiobarbituric acid reactive substances (TBARS), lipid peroxidation, and superoxide dismutase (SOD, antioxidant enzyme) were measured by using assay kits from the Biodiagnostic Company for Laboratory Services according to the given instructions. The measured liver tissue homogenates included reduced glutathione, glutathione reductase, catalase, SOD activities, and MDA as a marker of cell death in liver disease. According to Jafari and Rabbani, Hepatic DNA fragmentation was measured depending on colorimetrical quantitation upon staining with diphenylamine. This study was approved by the Scientific Induction Ethics Committee of Damanhour University, and the guidelines for the humane care of animals were applied.

#### Transmission electron microscope examination

By using 6.25% cacodylate buffer glutaraldehyde, all specimens from the liver and gills were fixed, followed by 1% osmium tetraoxide. After that, dehydration took place and the specimens were embedded in polyethylene capsules containing the embedding mixture: Epon mixture and hardener. Ultra-thin sections were prepared and stained with Uranyl acetate and lead citrate (Ali et al., 2014).

## Results and discussion

### Nanoparticles characterization

**FIGURE 1:**
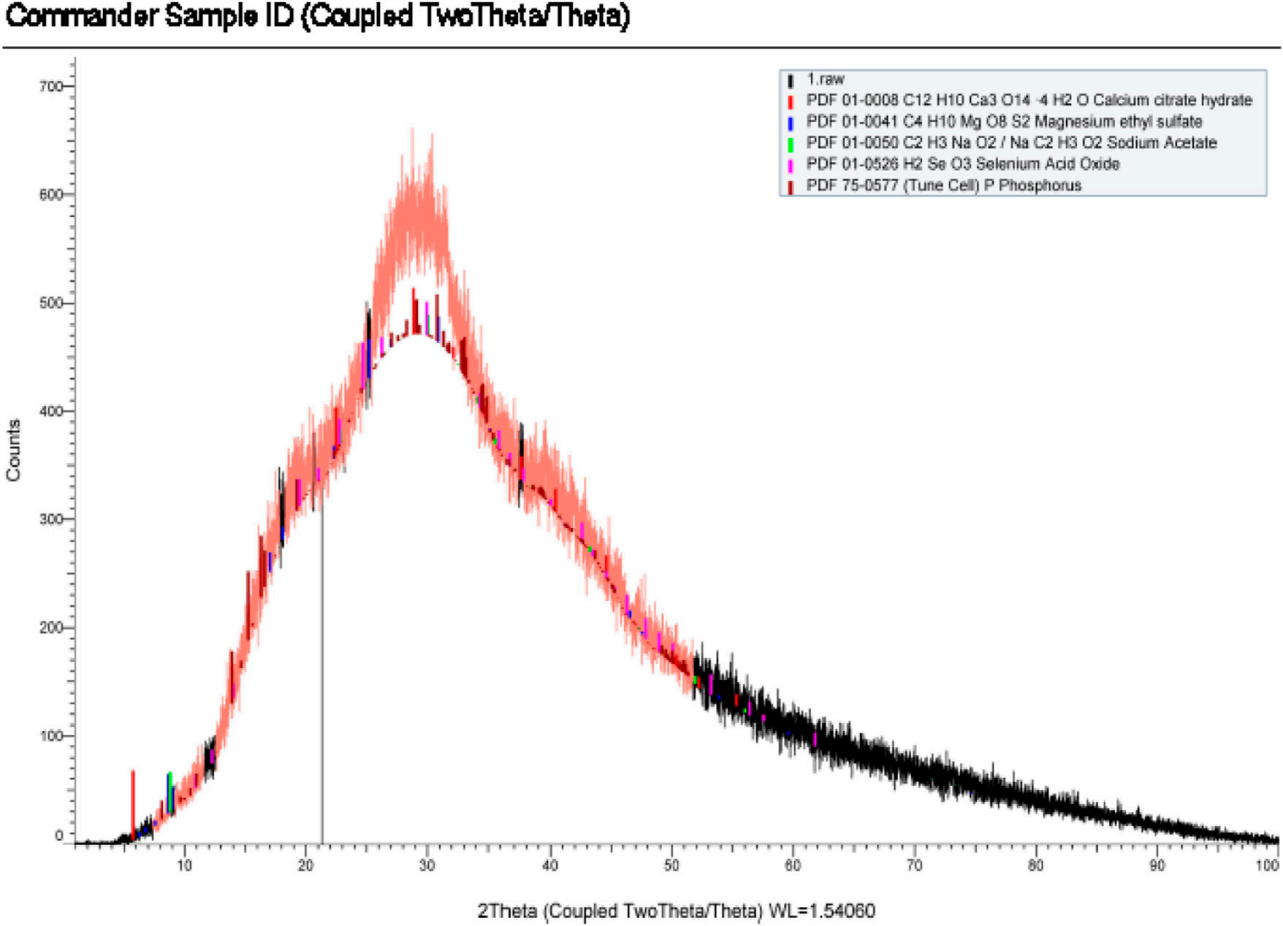
PoSeNPs produced some background noise in XRD pattern.

As illustrated in a previous study by Touliabah, et al. (2022), characterization was done for PoSeNPs through the mentioned parameters. A previous work showed that the zeta potential was charged with - 24.0 ± 5.62 mV (of 100% peak area) and 5.62 mV zeta deviation, with 0.0543 mS/cm conductivity. This zeta potential value indicates that these selenium nanoparticles have higher colloidal stability in an aqueous solution, which is most likely due to stronger repulsion behavior between single particles in a polar solution, i.e., water. This may be accomplished through the use of highly repulsive and attractive forces between nanoparticles and the presence of a negative charge on the surface of nanoparticles, which induces repulsion between them, resulting in nanoparticle stability (Mohamed and Bashir, 2012). The polydispersity index value (PDI = 0.094) indicates that PoSeNPs are polydispersed, indicating nanoparticle stability. This value is in the homogeneous size distribution range (0–1). When the PDI exceeds one, the size distribution homogeneity decreases, but increases as the PDI approaches zero. This result comes in line with Yedurkar et al. (2017). The phycosynthesized PoSeNPs had a maximum UV absorption peak of 350. XRD of *P. myrica*-mediated selenium nanoparticles exhibited the synthesis of crystalline spherical SeNPs with a size of 15.9 nm. Obtained patterns illustrate the main PoSeNPs characteristic peaks; Figure 1 shows PoSeNPs producing some background noise in an XRD pattern. From previous results, the FTIR of both *P. myrica* aqueous extract and PoSeNPs was with a high degree of similarity with a slight deviation in the detected absorbance bands, which proves the algal extract active group capping of SeNPs. This makes these nanoparticles more stable and effective (Kumari, et al., 2016).

**FIGURE 2:**
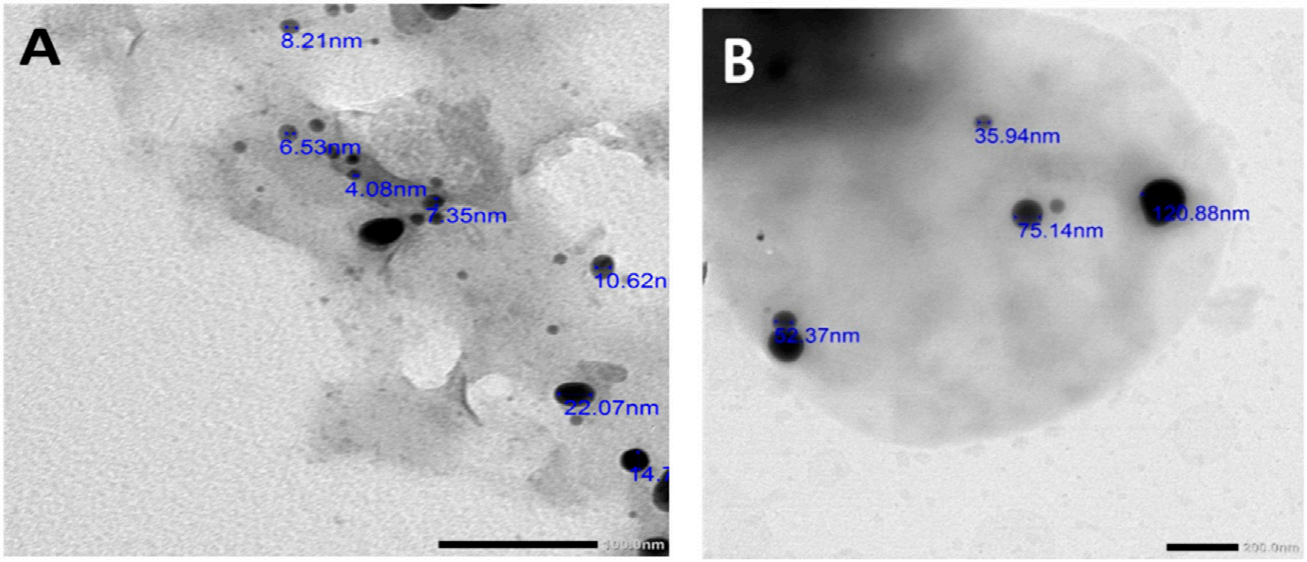
TEM imaging of PoSeNPS, showing different particle sizes and morphology **(A)** at scale bar (100 nm) and **(B)** at scale bar (200 nm).

**FIGURE 3:**
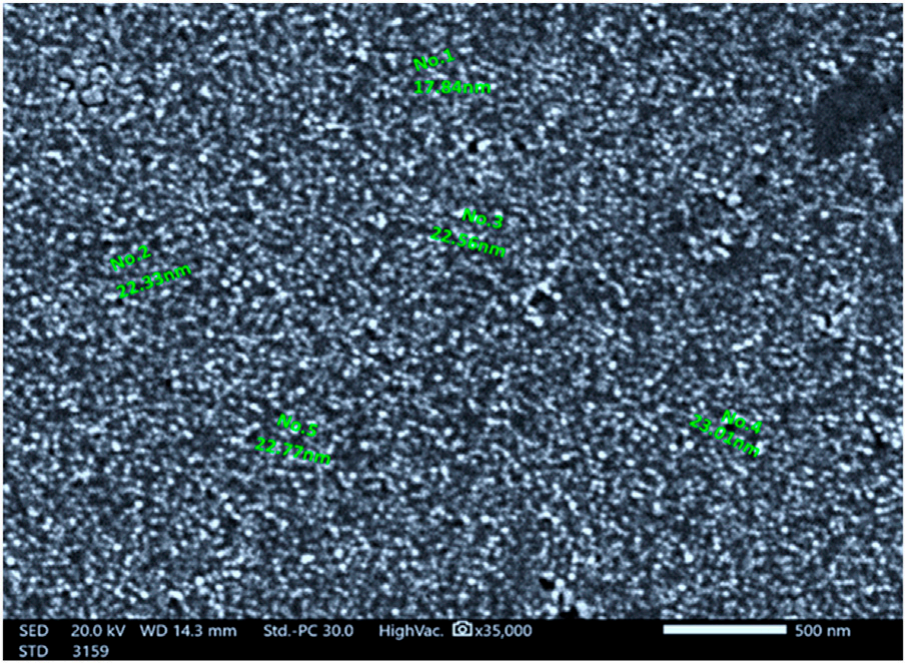
SEM imaging of PoSeNPS (500 nm) with different particle sizes.

**FIGURE 4:**
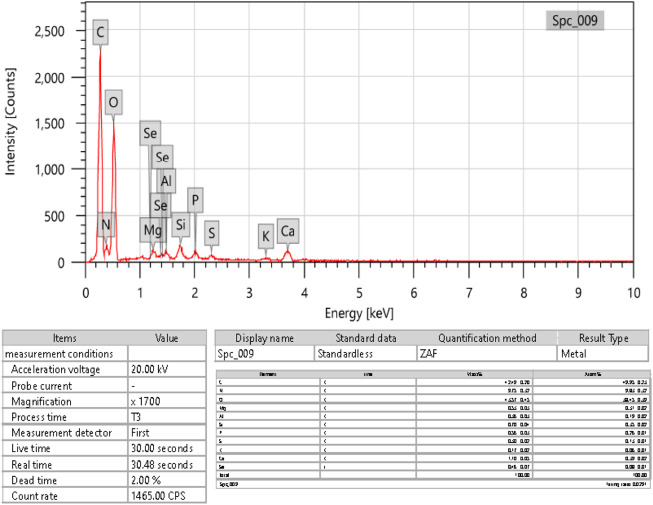
EDX analysis of PoSeNPS.

**FIGURE 5:**
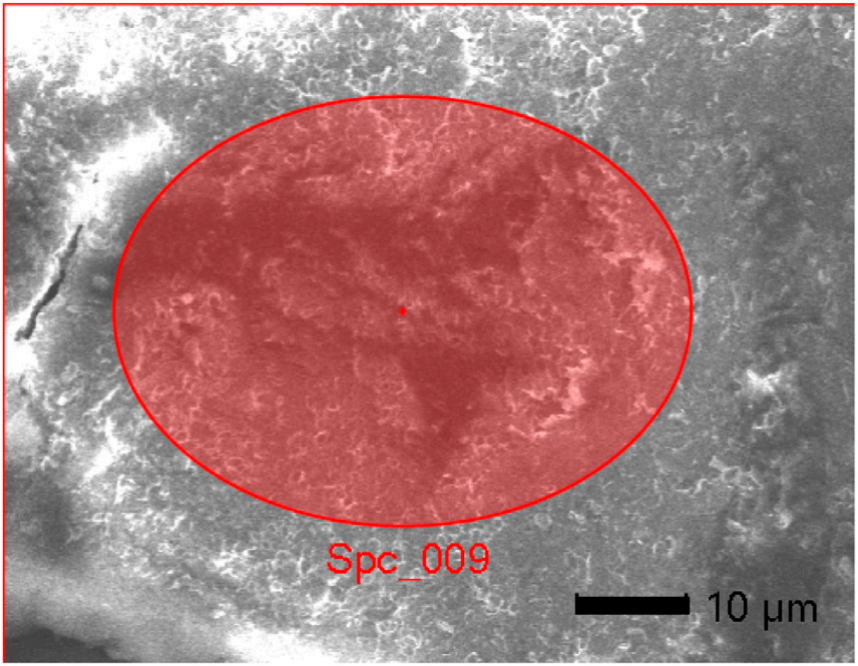
Field of EDX analysis of PoSeNPs.

Figures 2A, B show the TEM imaging of PoSeNPs at scale bars 100 and 200, respectively, with a range from 4.08 nm to 120.88 nm size and a smooth spherical shape. Such results go in harmony with El-Shanshoury et al. (2020), who synthesized selenium nanoparticles by using *Bacillus subtilis* and concluded that the produced nanoparticles were polydispersed and spherical, with particle sizes ranging from 31 nm to 193 nm. Meanwhile, Figure 3 shows SEM imaging that illustrates the oval spherical shaped of PoSeNPs with particle sizes ranging between 17.84 nm and 23.01 nm. Figures 4, 5 show the selenium elemental composition through EDX analysis. PoSeNPs had an atomic percent of 0.08 ± 0.01 and a mass percent of 0.46 ± 0.07 confirming the production of SeNPs. Other EDX peaks were detected including K, C, N, P, S, Si, Mg, Ca, Al, and O, with mass percentages of 0.17 ± 0.02, 42.49 ± 0.20, 9.75 ± 0.32, 0.56 ± 0.03, 0.30 ± 0.02, 0.70 ± 0.04, 0.53 ± 0.03, 1.10 ± 0.05, 0.36 ± 0.03 and 43.57 ± 0.45 atom percentages of 0.06 ± 0.01, 49.95 ± 0.23, 9.83 ± 0.32, 0.26 ± 0.01, 0.13 ± 0.01, 0.35 ± 0.02, 0.31 ± 0.02, 0.39 ± 0.02, 0.19 ± 0.02 and 38.45 ± 0.39, respectively.

### Virucidal effect of PoSeNPs

SeNPs have promising antiviral activity against many viruses (Kopel, et al., 2022). Table 1 illustrates the antiviral effects of PoSeNPs on the herpes simplex type-1 virus (HSV-1) when testing them at a non-cytotoxic maximum concentration of 50 μg/mL. PoSeNPs showed (++) moderate antiviral activity against HSV-1 with an inhibition of 35.25% ± 0.61%, and EC_50_ of 52.81 ± 1.03. The ratio of CC_50_—which was estimated in a previous study as 220.53 ± 6.89 μg/mL (Touliabah et al., 2022)—to EC_50_ was estimated to calculate the selectivity index ((SI) = 4.2), which illustrates if PoSeNPs had sufficient antiviral activity without normal cell toxicity (Al-Salahi et al., 2015). This index is considered a therapeutic index, showing that PoSeNPs can be considered active antiviral agents, which warrants further study since compounds with SI ≥ 2 are described as active. The virucidal effect of SeNPs against different viruses can occur due to the SeNPs direct binding to the glycoproteins in the viral envelope that do not allow the entrance of the virus into host cells, even though the action mechanism is still unknown (Lu, et al., 2008; Fayaz, et al., 2012). Makhlof et al. (2022) detected the antiviral activity of selenium nanoparticles synthesized by *Ulva lactuca* aqueous extract against HAV HM175 by using an MTT assay and found that USeNPs have good antiviral activity (+++) against the tested virus. SeNPs decreased the fragmentation of HepG2-infected cell lines DNA; the tail length particularly exhibited an eight-fold increase, and the tail DNA (%) showed a 3.7-fold increase in the HepG2 cell lines infected with HBV (Gad, et al., 2022). When the infection with HBV occurred in HeG2 cells and was then treated with SeNPs, DNA damage decreased (Gad, et al., 2022). Baram-Pinto et al. (2010) reported that AuNPs inhibited the spread and attachment of HSV-1.

**TABLE 1:** Virucidal effect of PoSeNPS upon HSV-1.

Sample name	MNCC (µg/mL)	Antiviral effect on HSV-1 (%) tested at MNCC	Antiviral effect on HSV-1 (Qualitative)^a^	Antiviral efficiency		
				EC_50_	CC_50_	SI
**PoSeNPs**	**50**	**35.25 ± 0.61**	++	52.81 ± 1.03	220.53 ± 6.89	4.2
**Acyclovir**	**20**	**90.73 ± 3.24**	**++++**	3.08	143.59	46.62
**Reference drug**						

^a^ Where: (++): Moderate antiviral activity (25-<50%) (++++): Excellent antiviral activity (75%-100%).

### Anticancer activity

#### *In vitro* studies

##### Cytotoxic evaluation of PoSeNPs against HCT-116

Colon cancer is considered a hazardous disease with high mortality and prevalence rates (Akanksha, et al., 2022). Selenium nanoparticles exhibit a considerable degree of specificity in cancer growth inhibition. This was confirmed by the suppression of the growth of colon cancer cells *in vitro* and *in vivo* through oral administration in a CT26 mouse infected with a colon cancer model. Moreover, SeNPs can trigger the generation of ROS in HT29 cancer cells, which can cause induction of apoptosis. However, in cancer, the metabolism of Se is altered, although how tumorigenesis, invasiveness, and malignancy impact this process is still unclear (Spyridopoulou, et al., 2021; Shimada, et al., 2022).

**FIGURE 6:**
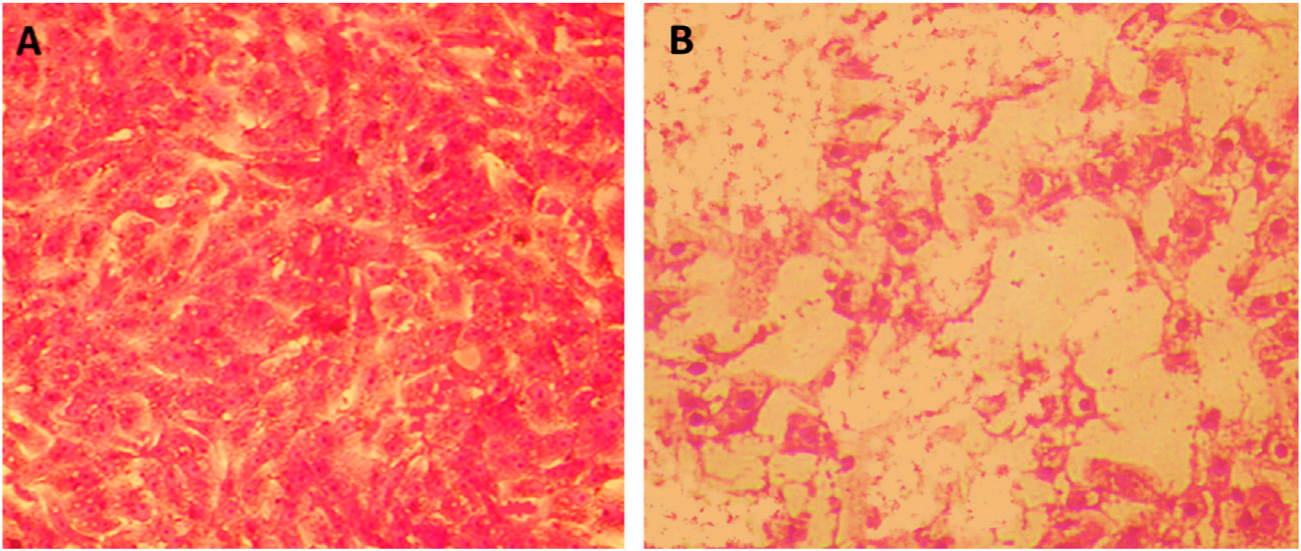
Cytotoxicity of PoSeNPS toward HCT-116 cell line. **(A)** is the control HCT-116 without PoSeNPS, and **(B)** is HCT-116 at a 50 μg/mL concentration.

Table 2 and Figures 6A, B showed the cytotoxicity of PoSeNPs with colon carcinoma cell line (HCT-116) with IC_50_ = 14.8 ± 0.57 μg/mL; the phycosynthesized nanoparticles showed a promising inhibitory effect toward HCT-116 cell line and their action increased in a concentration-dependent manner. PoSeNPs were used at concentrations of 0–50 μg/mL, the lowest viability of 19.43% ± 0.02%, with the highest inhibition of 80.57%, recorded at the highest nanoparticle concentration (50 μg/mL). Meanwhile, the highest viability of 100% and 93.89% ± 0.53%, with the lowest inhibition of 0% and 6.11% ± 0.22% recorded control and the lowest nanoparticle concentration of 1.56 μg/mL, respectively. The SeNPs anticancer mechanism is still not understood completely. However, some authors have suggested some mechanisms, such as the penetration of nanoparticles into cancer cells, the inhibitory effect of SeNPs on the activity of some cancer-induced enzymes such as epidermal growth factor receptor (EGFR), the controlling effect of SeNPs on ROS production, and trigger of autophagy and stimulation of cancer cells apoptosis (Menon, et al., 2018; Gao, et al., 2020; Makhlof, et al., 2022). Although all the previous mechanisms were suggested, additional new mechanisms are needed for destroying cancer cells. Thus, innovative treatment approaches need to be developed.

**TABLE 2:** Inhibitory activity of PoSeNPS against colon carcinoma cells (HCT-116) with IC_50_ = 14.8 ± 0.57 μg/mL.

Sample conc. (µg/mL)	Viability %	Inhibitory %
0	100	0
1.56	93.89 ± 0.53	6.11
3.125	85.14 ± 0.22	14.86
6.25	74.93 ± 1.65	25.07
12.5	53.12 ± 1.34	46.88
25	36.25 ± 1.03	63.75
50	19.43 ± 0.02	80.57

The data are expressed in the form of mean ± SD.

##### Cytotoxic evaluation of PoSeNPs against EAC cells

**FIGURE 7:**
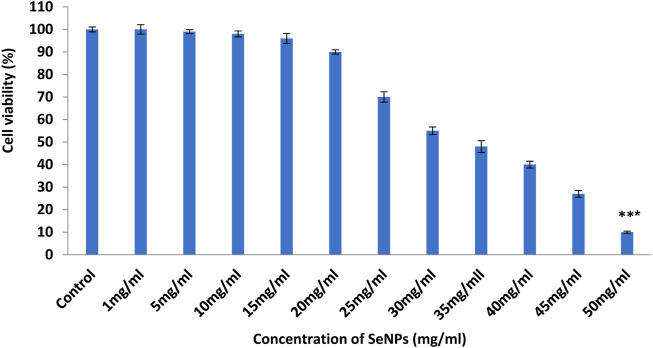
Effect of PoSeNPs on cell viability of EAC cells determined by MTT assay. Cell viability of EAC cells treated with different concentrations (ranging from 1 to 50 *m*g/mL) of SeNPs. Data are expressed as the mean ± SD, *** indicate very high significant difference as compared with the control treatment (*p* < 0.001).

As illustrated in Figure 7, EAC viability decreased significantly (*p* < 0:05) during the PoSeNPs treatment at concentrations from 25 mg/mL to 50 mg/mL. However, SeNPs at doses from 25 mg/mL to 50 mg/mL showed no inhibition effect on EAC cell line growth (*p* > 0.05). The cytotoxic responses of EAC cells treated with 1–50 mg/mL of SeNPs were evaluated by an MTT assay. This assay showed that SeNPs tested at a concentration of up to 20 μg/mL did not produce any cytotoxicity (*p* > 0.05 for each). However, the SeNPs at 25, 30, 35, 40, 45, and 50 mg/mL showed concentration-dependent cytotoxicity. The cell viability decreased to 70%, 55%, 48%, 40%, 27%, and 10% at 25, 30, 35, 40, 45, and 50 mg/mL SeNPs, respectively. Selenium nanoparticles were mainly located in the mitochondria of tumor cells and induced apoptosis in tumor cells. The LNT in SeNPs was involved in caveolae-mediated endocytosis through the interaction between toll-like receptor-4 (TLR4) and caveolin 1 (CAV1). Furthermore, the SeNPs in the endocytic vesicles can enter the mitochondria through the mitochondrial membrane fusion pathway, which was mediated by TLR4/TNF receptor-associated factor 3 (TRAF3)/mitofusin-1 (MFN1) protein complex (Liu, et al., 2022).

#### *In vivo* studies

##### Tumor volume

**FIGURE 8:**
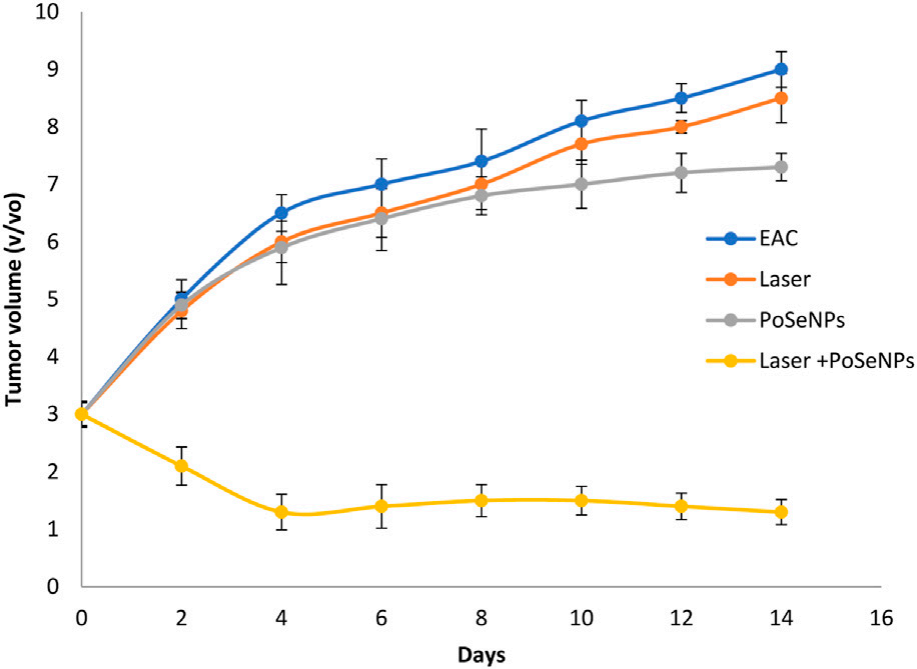
Effect of different treatments showing change in the relative tumor volume.

On the 14th day, we begin to determine the tumor volume change as shown in Figure 8. The tumor grows consistently in control (EAC), the laser alone, PoSeNPs alone, and the combination of laser and PoSeNPs groups. The relative tumor volumes (V/Vo) on the 14^th^ day are 9.12 ± 0.22 in the control (EAC) group, 8.5 ± 0.24 in the laser group, and 7.3 ± 0.31 in the PoSeNPs group. The results in this study illustrated that in the case of the treatment with laser irradiation alone or PoSeNPs injection alone, the tumor development did not exhibit remarkable reduction. Meanwhile, in the case of laser + PoSeNPs, there was a very high significant decrease in tumor volume. This is an indicator of tumor shrinking and disappearance. Laser + PoSeNPs show high-performance photothermal therapy and are promising for further biomedical applications.

##### Effect on mortality rate and survival curve

**FIGURE 9:**
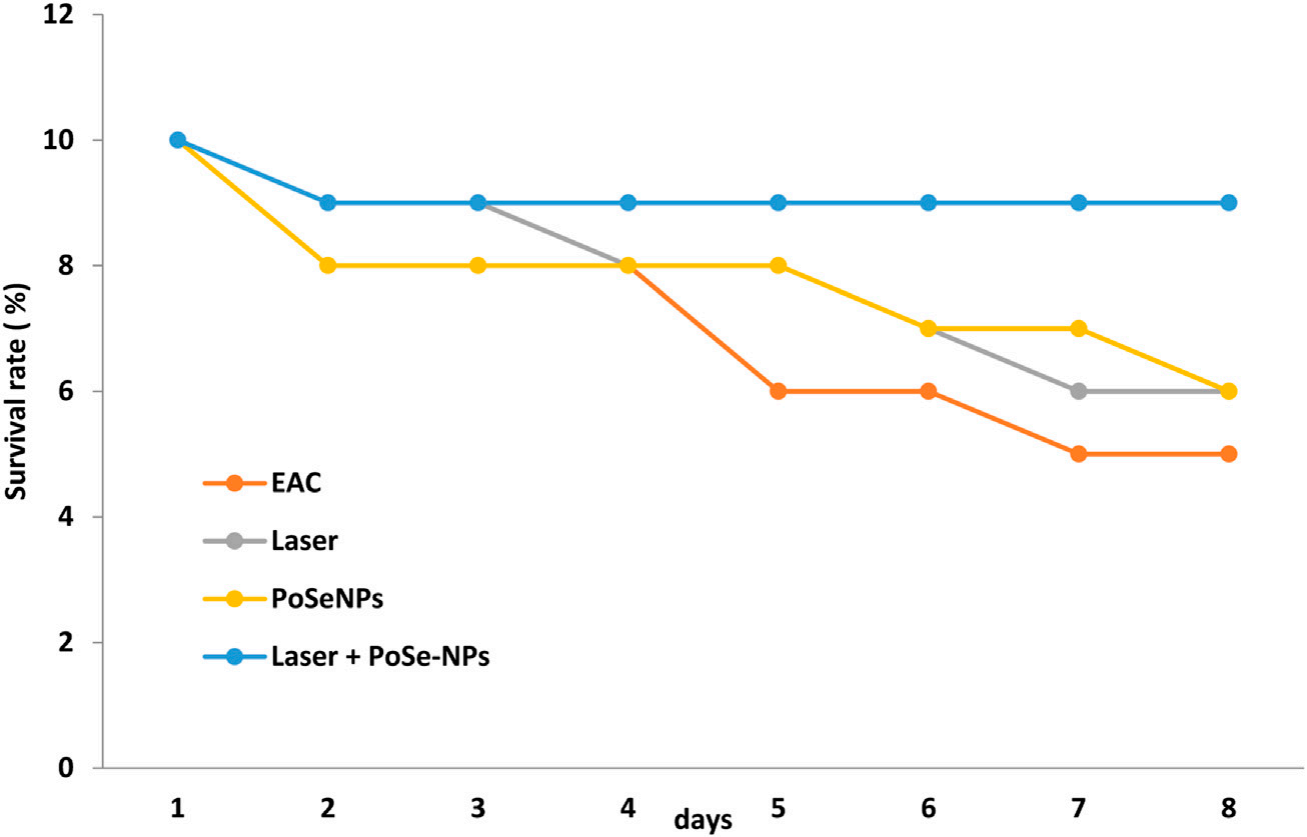
Survival rate of the mice after different treatments.

Mortality rate is represented in Table 3; the mortality percentage of mice will be 10% when mice bearing tumors receive PoSeNPs, and are then irradiated by laser therapy. This is an indicator of the improvement in the biological system of mice. It was observed that combined therapy inhibits cancer cell proliferation. The survival rates of mice after different treatments are represented in Figure 9.

**TABLE 3:** Effects of PoSeNPs (50 mg/kg), radiation therapy, and their combination on the survival of EAC tumor-bearing mice.

Groups	Number of mice	Survivors/total mice	Mortality% (%)
NEAC	10	10/10	0
EAC	10	5/10	50
Laser	10	6/10	40
PoSeNPs	10	6/10	40
Laser + PoSeNPs	10	9/10	10

##### Liver and kidney function tests

Table 4 reveals the increment in the plasma AST, ALT, Urea, and creatinine of tumor-bearing mice, which explains different toxicities that occurred because of the development of cancer, and thus the catabolic effect of the tumor. In addition, the result exhibits kidney and liver toxicity produced in combination with tumor development, which can result from excessive stress due to reactive oxidative, which in turn causes oxidative damage that leads to liver and kidney dysfunction. No significant difference was observed in the case of the Laser + PoSeNPs group in ALT, AST, serum urea, and creatinine. The results of this *in vivo* study illustrated that the Laser + PoSeNPs treatment is more effective than the treatment by PoSeNPs alone or laser therapy alone. SeNPs exhibited excellent bioavailability because of low toxicity, strong ability to adsorb, and catalytic efficiency. Laser + PoSeNPs treatment against Ehrlich ascites carcinoma cells improves kidney and liver function tests, and decreases cancer cell metastases and apoptosis. Selenium nanoparticles can change many cell cycle-related genes and apoptosis, which in turn prevents cancer. Table 5 shows a decrease in SOD activity in mice bearing tumors. This decline in SOD activity is an indicator of the loss of mitochondria. The inhibition activity of SOD is also reported which occurred as a result of tumor growth decrement. (Choudhury et al., 2008).

**TABLE 4:** Liver and kidney function test.

Groups	ALT (u/l)	AST (U/L)	Urea (mg/dL)	Creatinine (mg/dl)
NEAC	54 ± 2.53	90 ± 2.11	31.26 ± 0.32	0.83 ± 0.42
EAC	86 ± 1.22***	133 ± 0.95***	40.13 ± 0.63***	0.96 ± 0.21***
Laser	75 ± 2.41**	116 ± 2.34***	35.34 ± 0.87*	0.92 ± 0.22***
PoSeNPs	71 ± 0.81**	109 ± 2.11**	32.36 ± 1.32*	0.89 ± 0.15**
Laser + PoSeNPs	50 ± 0.54 ^**NS**^	93 ± 1.37 ^**NS**^	30.68 ± 0.74 ^**NS**^	0.80 ± 0.17^NS^

Each value is the mean ± SEM., Non-significant (N.S): *p* > 0.05; Significant (S): *p < 0.05; highly significant (HS): **p < 0.01; very highly significant (VHS): ***p < 0.001 from NEAC.

**TABLE 5:** Effect of different treatment on SOD activity in blood, liver, and tumor tissue.

	SOD activity		
Groups	Blood (u/mL)	Liver tissue (u/g tissue)	Tumor tissue (u/g tissue)
NEAC	4.22 ± 0.15	5.19 ± 0.27	8.42 ± 0.07
EAC	2.54 ± 0.23***	4.28 ± 0.09**	6.30 ± 0.84***
Laser	4.31 ± 0.54**	7.53 ± 0.08***	7.36 ± 0.14*
PoSeNPs	4.52 ± 0.26**	7.75 ± 0.36***	7.77 ± 1.3*
Laser + PoSeNPs	4.11 ± 0.34 ^**NS**^	5.43 ± 0.11 ^**NS**^	7.98 ± 0.43^NS^

Each value is the mean ± SEM., Non-significant (N.S): *p* > 0.05; Significant (S): *p < 0.05; highly significant (HS): **p < 0.01; very highly significant (VHS.

### Histopathological examination

#### In tumor tissue

**FIGURE 10:**
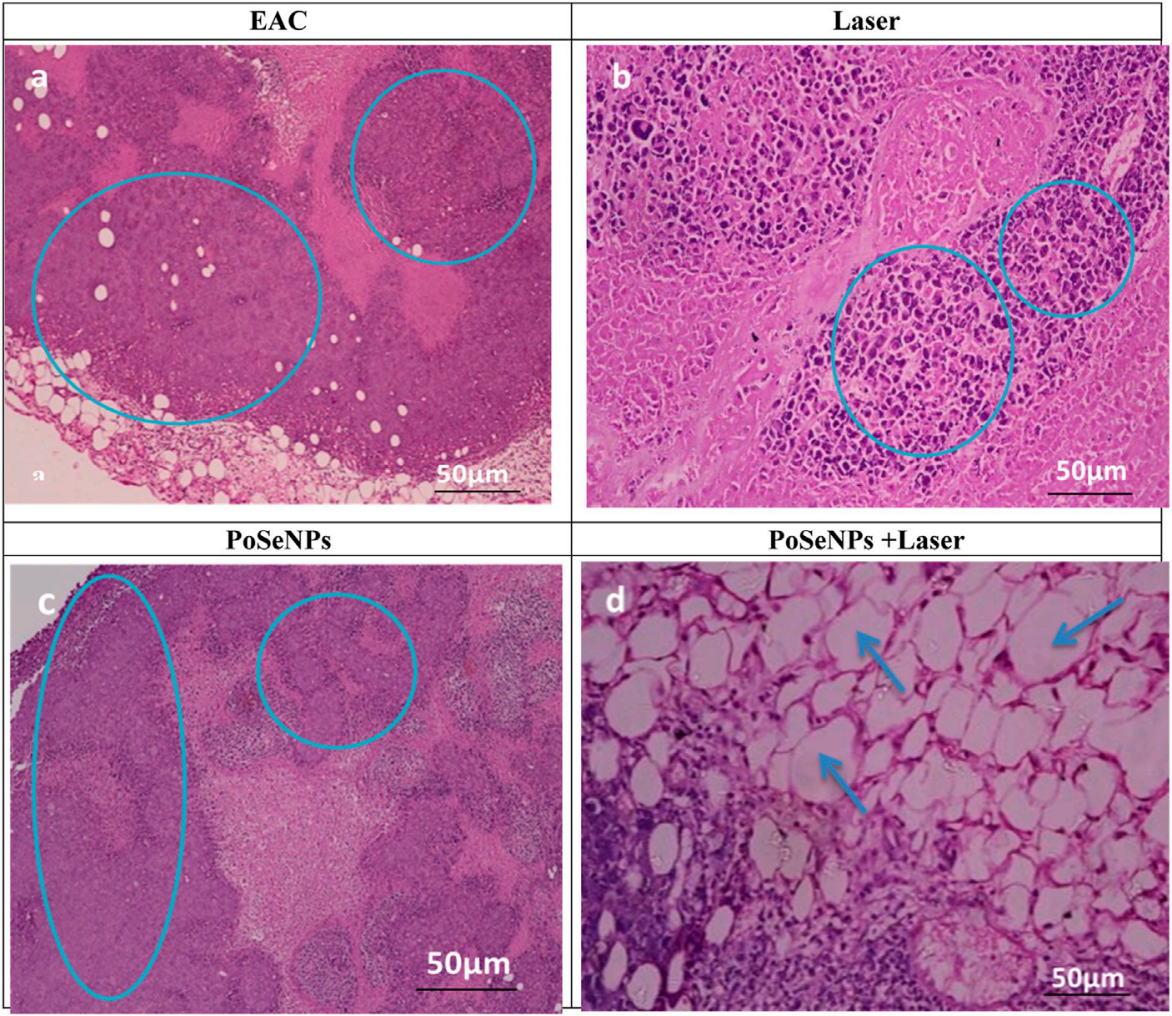
Histological analysis of tumor injury stained with hematoxylin and eosin (H&E) (Scale bare:50 µm). **(A)** Tumor without treatment showing inflammatory cells (blue circle) **(B)** Tumor irradiated with laser showing inflammatory cells (blue circle) **(C)** injected tumor with PoSeNPs showing inflammatory cells (blue circle) **(D)** injected tumor with PoSeNPs then irradiated by laser beam showing necrosis cells (blue arrow).

By using hematoxylin and eosin (H&E) stain, tumor analysis was conducted as shown in Figure 10, displaying histological analysis of tumor injury stained with hematoxylin and eosin (H&E) (Scale bare:50 µm). Figure10A shows tumor without treatment, which exhibits inflammatory cells (blue circle). Both the tumor irradiated only with laser (Figure 10B: blue circle) and tumor injected with PoSeNPs (blue circle, Figure 10C) show inflammatory cells. However, tumor injected with PoSeNPs then irradiated by laser beam shows necrosis cells (blue arrow, Figure 10D). There are no remarkable changes pathologically in each of the following groups: control, the laser alone, and PoSeNPs alone. But significant necrosis was observed in the case of laser plus PoSeNPs group. Tumor vessels also destroy cell membrane and necrosis and cause pyknosis to occur in the nucleus region.

These results demonstrate that laser irradiation alone or PoSeNPs injection alone does not affect tumor development. However, treatment by a combined therapy (PoSeNPs plus laser) leads to tumor inactivation, tumor shrink, and black scars left in the tumor site, and the tumor disappears due to thermal damage with the appearance of smooth scars on the tumor original site—which reveals that PoSeNPs are an effective photodynamic thermal therapy agent for *in vivo* cancer therapy. PoSeNPs plus Laser group shows massive necrosis in tumor cells with slight aggregations of malignant cells as indicated with a blue arrow. Therefore, PoSeNPs plus Laser treated group showed the most effective EAC tumor tissue destruction when compared to the control group (Jingxia et al., 2020).

#### In liver tissue

**FIGURE 11:**
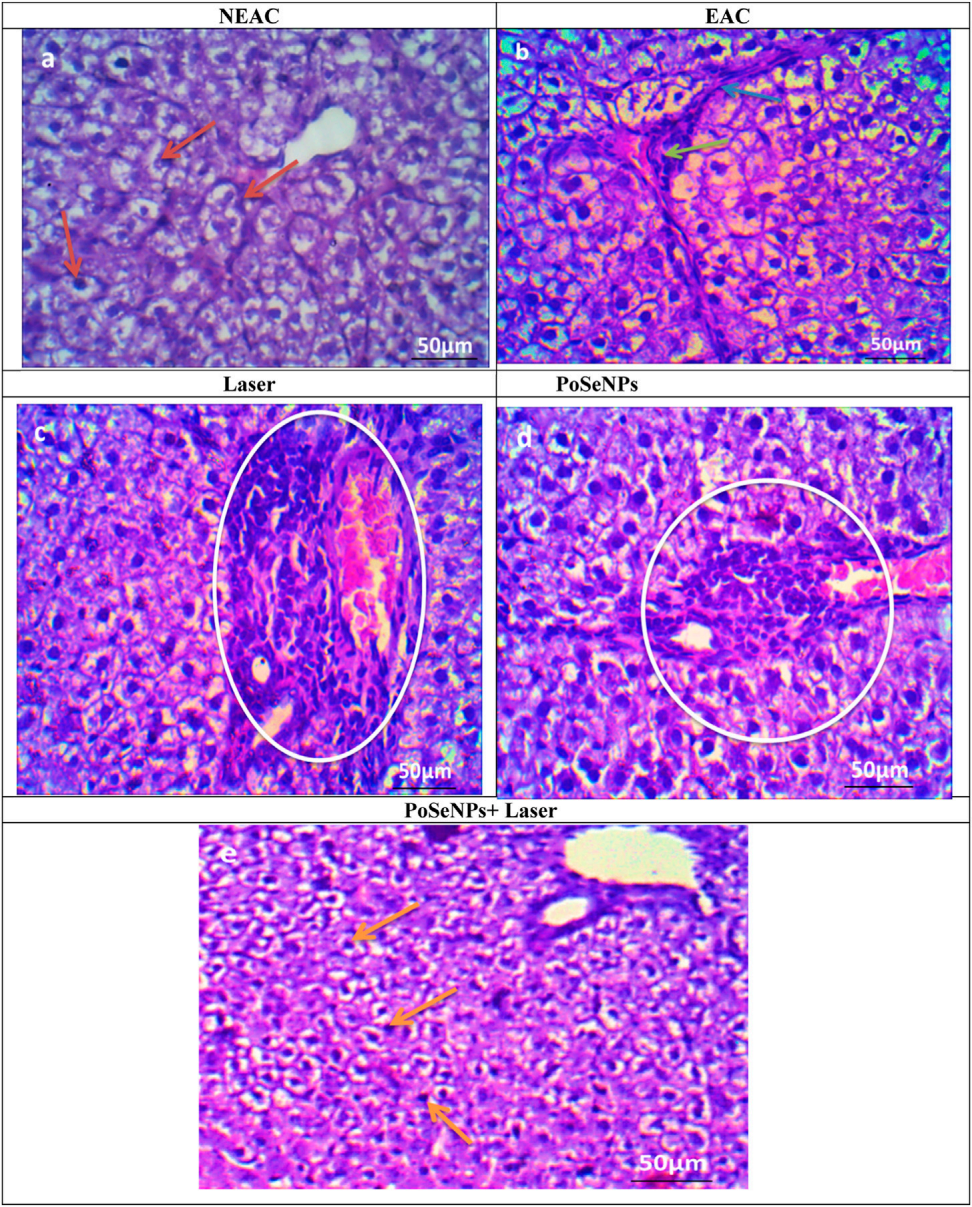
Photomicrographs of sections in liver stained by H&E **(A)** Liver of negative control group showing normal hepatic architecture (red arrow) (scale bare:50 µm). **(B)** Liver of mice bearing Ehrlich carcinoma (positive control group) showing birding portal fibrosis (blue arrow) and focal aggregation of neoplastic cells (green arrow) (scale bare:50 µm). **(C)** Liver of tumor bearing mice treated only with laser irradiation showing birding portal inflammation (White circle) (scale bare:50 µm).). **(D)** Liver of tumor bearing mice treated only with PoSe-NPs showing birding portal inflammation (White circle) (scale bare:50 µm). **(E)** Liver of tumor bearing mice treated with PoSe-NPs then irradiated with laser radiation, showing no inflammatory cells and normal hepatic cells (orange arrow) could be detected (scale bare:50 µm).

There was a histopathological examination of liver sections stained by H&E of the normal liver with normal hepatic cells (red arrow, Figure 11A) on EAC-inoculated animals. Hepatocytes showed hydropic changes with pyknotic nuclei and necrosis, with the spread of various neoplastic foci within the liver when compared with the control liver, which distributed as a focal mass as indicated with the blue arrow and focal aggregation of neoplastic cells (green arrow) (Figure 11B). This could be due to ascetic fluid accumulation inside the peritoneal cavity—the site where cell proliferation occurs, then moves to invade internal organs. It also increases the deposition of connective tissue that surrounds the liver’s central veins. The presence of collagen can affect the blood supply to liver cells and reduce the exchange of metabolites, thus causing necrosis. The central vein with lymphocytes around it induces an increase in the glycogen content of liver cells. It also induces a decrease in hepatic glutathione content (GSH) and superoxide dismutase (SOD) activity. This reduction in GSH and SOD activity can change antioxidant defenses, resulting in enhanced oxidation due to ROs increment and accumulation and reduction of liver tissue antioxidants (Anna et al., 2021).

The liver section of laser alone (Figure 11C) and PoSeNPs (Figure 11D) in treated mice showed moderate aggregation of neoplastic cells (white circle, Figure 11C) and revealed the abnormal appearance of most hepatocytes, vacuolated cytoplasm with pyknotic nuclei (white arrow, Figure 11D), and necrosis which leads to hydropic degeneration of the cytoplasm. Inflammatory cells also appeared around the central vein. These changes could be due to the degeneration of mitochondrial and cytoplasm disorganization (Mohamed et al., 2022).

In contrast, for PoSeNPs plus laser group, as shown in Figure 11E, no inflammatory cells can be detected, while hepatic lobules have few hepatic cells. It also showed the least focal aggregation of neoplastic cells. The cells were generally less vacuolated, and collagen fibers were seen around the central vein (orange arrow, Figure 11E). The combined treatment by radiation therapy after injection by PoSeNPs appears to be useful in reducing both hepatic and oxidative stress damage, and also reducing tumor count and fluid volume (Cláudio et al., 2021).

**FIGURE 12:**
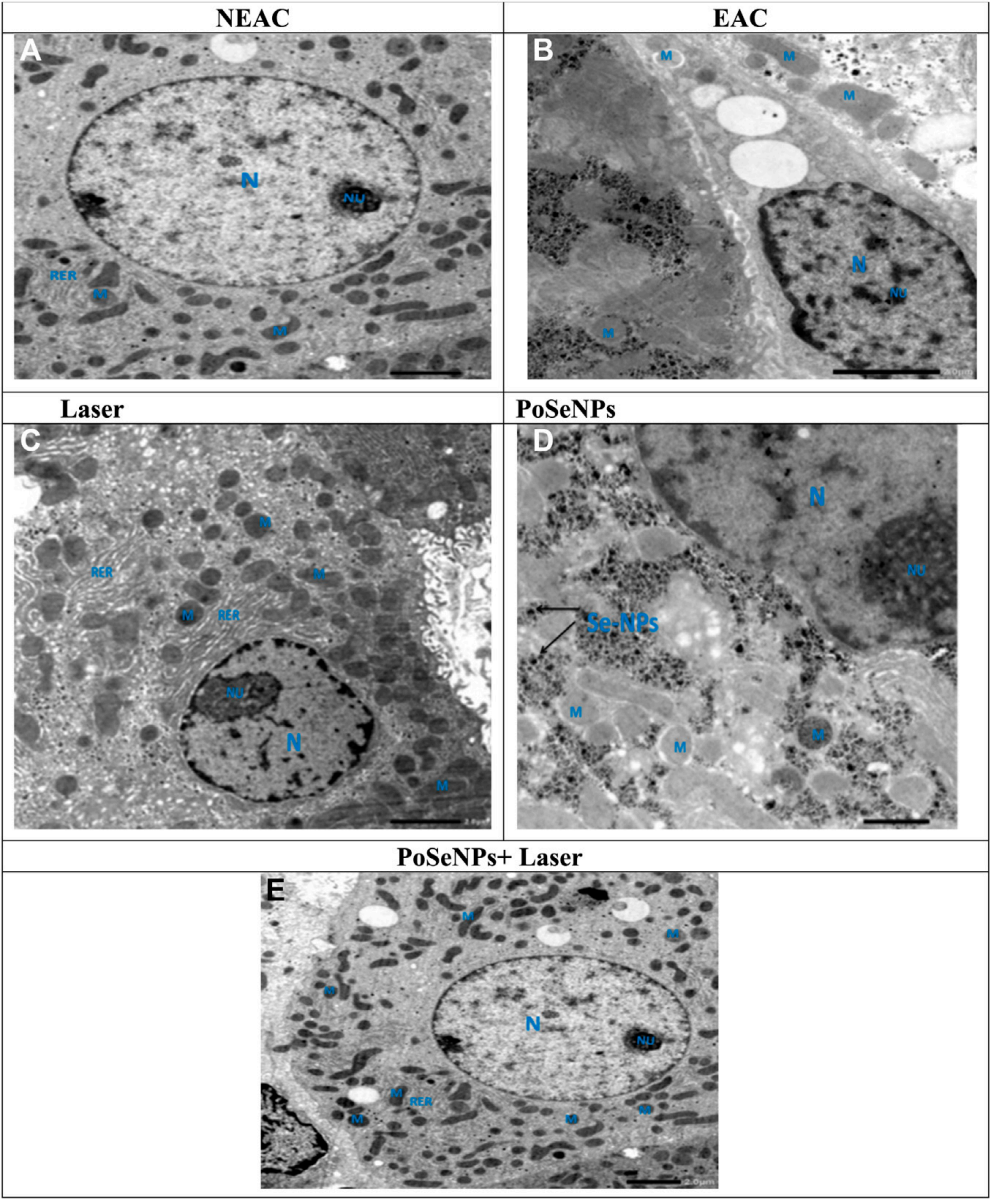
Transmission electron microscope photomicrographs of liver tissue from NEAC, EAC mice treated with nano, laser, and Nano + laser. A NEAC Control group **(A)** showing the cytoplasm of the hepatocyte containing rounded euchromatic nucleus (N) with prominent nucleolus (Nu), oval mitochondria (M), and rough endoplasmic reticulum (RER). EAC group **(B)** showing hepatocytes contained nuclei with clumped dense chromatin the nucleus appeared irregular in shape (N), and slightly swollen mitochondria (M). Laser group **(C)** showing swollen mitochondria (M), and RER dilatation (RER). PoSeNPs group **(D)** showing polymorphic mitochondria (M) with dense granules, maricets, and deposition of aggregated PoSeNPs, and the nucleus appeared irregular in shape (N). PoSeNPs + laser Group **(E)** showing nearly normal architecture of hepatocytes. The cytoplasm contained rounded euchromatic nucleus (N) with prominent nucleolus (Nu), the mitochondria (M), and (RER) retain its normality.

Figure 12 shows TEM imaging of liver tissue from NEAC, EAC mice treated with PoSeNPs, laser, and PoSeNPs plus laser. The NEAC Control Group (A) showed a rounded euchromatic nucleus (N) in hepatocyte cytoplasm with prominent nucleolus (Nu), oval mitochondria (M), and rough endoplasmic reticulum (RER). EAC Group (B) showed hepatocytes containing nuclei with clumped dense chromatin that (N) appeared irregular in shape and slightly swollen (M). Laser Group (C) showed swelling (M) and dilatation (RER). PoSeNPs Group (D) showed polymorphic (M) with dense granules, maricets, and deposition of aggregated SeNPs, and (N) appeared irregular in shape. Nano + Laser Group (E) showed nearly normal hepatocytes with normal architecture. The rounded euchromatic (N) with prominent (Nu) was also observed in the cytoplasm with the presence of (M), and (RER) retained its normality.

As described previously, the control liver cells showed cytoplasm with numerous (M) RER, Golgi apparatus, few fat lipid globules, and glycogen granules. Meanwhile, a round normal (N) occurred with a central (Nu) in location and dispersed granular chromatin. A NEAC Group (A), which represents control, shows rounded euchromatic (N) within the hepatocyte cytoplasm with prominent (Nu), oval (M), and (RER) (Figure 12A). EAC mice were treated with nano, laser, and Nano + laser. EAC Group (Figure 12B) showed hepatocytes containing nuclei with clumped dense chromatin that (N) appeared irregular in shape and slightly swollen (M). Laser Group (Figure 12C) showed swelling (M) and RER dilatation. PoSeNPs Group (Figure 12D) showed polymorphic (M) with dense granules, maricets, and deposition of aggregated SeNPs, and (N) appeared irregular in shape. PoSeNPs plus laser Group (Figure 12E) showed hepatocytes architecture with nearly normal appearance. Rounded euchromatic (N) with prominent (Nu) was observed in the cytoplasm with the presence of (M), and (RER) retained its normality (Mannucci et al., 2020).

## Conclusion

SeNPs-*in vivo* alone and in combination with laser therapy with 2 mW power-revealed a potent therapy effect against Ehrlich ascites carcinoma. Therefore, PoSeNPs act as an anticancer agent, and can be considered the right photothermal choice for various types of cancer cells treated with high efficiency and low toxicity on normal cells. They are promising for further biomedical applications as an antiviral agent. The combined therapy exhibited a highly significant decrease in tumor volume, massive tumor cell necrosis, shrinking, and disappearance. It also showed improvement in liver TEM, histology, liver enzymes: ALT and AST, and kidney function: urea and creatinine.

## Plant material

The brown alga *Polycladia myrica* was collected and identified by Prof. Fekry Ashour Murad, in the National Institute of Oceanography and Fisheries (NIOF) Suez branch.

## Data Availability

The original contributions presented in the study are included in the article/supplementary material, further inquiries can be directed to the corresponding author.
